# Isoprene Aerosol Growth in the Upper Troposphere: Application of the Diagonal Volatility Basis Set to CLOUD Chamber Measurements

**DOI:** 10.1021/acsestair.5c00106

**Published:** 2025-09-15

**Authors:** Nirvan Bhattacharyya, Brandon Lopez, Jenna DeVivo, Douglas M. Russell, Jiali Shen, Eva Sommer, João Almeida, Antonio Amorim, Hannah M. Beckmann, Mattia Busato, Manjula R. Canagaratna, Lucia Caudillo, Anouck Chassaing, Theodoros Christoudias, Lubna Dada, Imad El-Haddad, Richard C. Flagan, Hartwig Harder, Bernhard Judmaier, Milin Kaniyodical Sebastian, Jasper Kirkby, Hannah Klebach, Markku Kulmala, Felix Kunkler, Katrianne Lehtipalo, Lu Liu, Bernhard Mentler, Ottmar Möhler, Aleksandra Morawiec, Tuukka Petäjä, Pedro Rato, Birte Rörup, Samuel Ruhl, Wiebke Scholz, Mario Simon, António Tóme, Yandong Tong, Jens Top, Nsikanabasi Silas Umo, Rainer Volkamer, Jakob Weissbacher, Doug R. Worsnop, Christos Xenofontos, Boxing Yang, Wenjuan Yu, Marcel Zauner-Wieczorek, Imad Zgheib, Jiangyi Zhang, Zhensen Zheng, Xu-Cheng He, Dominik Stolzenburg, Siegfried Schobesberger, Joachim Curtius, Neil M. Donahue

**Affiliations:** † Center for Atmospheric Particle Studies, 6612 Carnegie Mellon University, Pittsburgh, Pennsylvania 15213, United States; ‡ Department of Chemistry, 6612 Carnegie Mellon University, Pittsburgh, Pennsylvania 15213, United States; § Department of Chemical Engineering, 6612 Carnegie Mellon University, Pittsburgh, Pennsylvania 15213, United States; ∥ Institute for Atmospheric and Environmental Sciences, Goethe University Frankfurt, 60438 Frankfurt am Main, Germany; ⊥ Institute for Atmospheric and Earth System Research/Physics, Faculty of Science, 3835 University of Helsinki, 00014 Helsinki, Finland; # Helsinki Institute of Physics, 3835 University of Helsinki, 00014 Helsinki, Finland; ∇ 30531 CERN, The European Organization for Nuclear Research, 1211 Geneva, Switzerland; ○ Faculty of Physics, University of Vienna, 1010 Wien, Austria; ◆ CENTRA and Faculdade de Ciências da Universidade de Lisboa, 1749-0016 Lisboa, Portugal; ¶ Department of Environmental Physics, University of Tartu, 50090 Tartu, Estonia; & Aerodyne Research, Inc., Billerica, Massachusetts 01821, United States; ● Department of Environmental Science, Stockholm University, 10691 Stockholm, Sweden; ◊ Climate and Atmosphere Research Centre, The Cyprus Institute, Nicosia 1645, Cyprus; ▲ PSI Center for Energy and Environmental Sciences, Paul Scherrer Institute, 5232 Villigen PSI, Switzerland; □ Division of Chemistry and Chemical Engineering, California Institute of Technology, Pasadena, California 91125, United States; ⊗ Max Planck Institute for Chemistry, 55128 Mainz, Germany; ¢ Institute for Ion Physics and Applied Physics, University of Innsbruck, 6020 Innsbruck, Austria; + Institute of Meteorology and Climate Research, Karlsruhe Institute of Technology, 76131 Karlsruhe, Germany; $ Finnish Meterological Institute, 00560 Helsinki, Finland; ∠ Instituto Dom Luiz, Universidade da Beira Interior, 6201 Covilhã, Portugal; € Department of Chemistry, University of Colorado Boulder, Boulder, Colorado 80309, United States; ¤ Cooperative Institute for Research in Environmental Sciences, University of Colorado Boulder, Boulder, Colorado 80309, United States; ¥ Tofwerk AG, 3645 Thun, Switzerland; ☼ IONICON Analytik GmbH, 6020 Innsbruck, Austria; ± Yusuf Hamied Department of Chemistry, University of Cambridge, Cambridge CB21TN, U.K.; ∞ Institute of Materials Chemistry, TU Wien, 1040 Vienna, Austria; †† Department of Technical Physics, University of Eastern Finland, 70211 Kuopio, Finland; ‡‡ Department of Engineering and Public Policy, 6612 Carnegie Mellon University, Pittsburgh, Pennsylvania 15213, United States

**Keywords:** atmospheric chemistry, particle growth, chamber measurements, volatility, isoprene

## Abstract

Isoprene oxygenated organic molecules (IP-OOM) can nucleate new particles in the upper troposphere. These particles may grow into cloud condensation nuclei and influence the clouds and climate. However, little is known about the individual species driving growth and whether they undergo condensed-phase reactions. We conducted isoprene oxidation experiments at 223 and 243 K in the CLOUD chamber at CERN. Gas-phase concentrations were measured with chemical ionization mass spectrometers (NO_3_^–^-CIMS, Br^–^-MION2-CIMS, and NH_4_^+^-CIMS). Growth rates from 8 to 20 nm were measured by a Neutral Cluster and Air Ion Spectrometer. Particle-phase composition was measured by a filter sampling chemical ionization mass spectrometer. We use the diagonal volatility basis set (dVBS) analysis framework to compare gas- and particle-phase measurements and assess species and processes influencing growth. We find that kinetically limited condensation of a few species dominates particle composition and growth. Particle-phase processes, including oligomerization and organonitrate hydrolysis, do not influence the early growth. dVBS growth rate predictions can explain 90% of the measured growth, dominated by kinetic condensation of low-volatility species. Our findings indicate that initial growth of IP-OOM particles under cold, low-acid conditions may be controlled and modeled by the kinetically limited condensation of low-volatility compounds.

## Graphical abstract

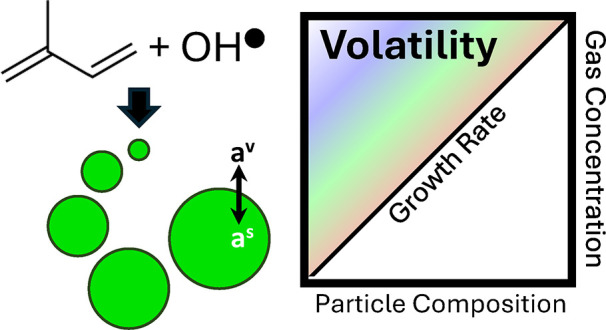

## 1 Introduction

Atmospheric particle growth is driven by condensation of vapors from the gas-phase to available aerosols.^1–3^ Like all phase changes, condensation is controlled by the activity of components in each phase and driven toward the equilibrium condition with equal activity in both phases and no net mass transfer. Particle-phase activity is proportional to the mass fraction of the compound in the particle-phase, which is bounded from 0 to 1, while gas-phase activity is proportional to the gas-phase concentration and ranges over many orders of magnitude. If an enormous supersaturation of gas-phase species is observed without sufficient particles present, then nucleation can occur. New particles provide a sink for the existing vapors and grow as compounds partition into the suspended phase. The relative influence on particle growth of nonreactive condensation compared to condensed phase reactions and reactive uptake is an open question for a wide range of inorganic and organic systems.

Isoprene is a key atmospheric biogenic volatile organic compound, with emissions of approximately 500 Tg annually.^4^ Previous studies focused on the planetary boundary layer have found that isoprene suppresses new particle formation in mixed α-pinene and isoprene systems while contributing to growth.^5,6^ However, recent aircraft measurements,^7^ experiments,^8^ and models^9^ have shown nucleation of isoprene oxidation products high above the Amazon following convective uplift. In particular, observations showed that condensable vapors were dominated by isoprene-derived organonitrates. According to the condensation theory discussed above, such species should predominate in particle-phase compositions during steady-state growth. However, it is possible that reactive uptake or particle-phase processing alters the particle composition and controls isoprene-derived particle growth. For example, gas-phase dimerization may be the primary path for initial nucleating organic clusters, but during growth, such dimers can be formed by oligomerization reactions in the suspended phase.^10^ For organonitrate isoprene oxidation products, hydrolysis may play a role in their fate after uptake to particles, transforming nitrate functionalities into alcohols:^11–13^

$${R-\mathrm{ONO}}_{2}+H_{2}O\to R-\mathrm{OH}+{\mathrm{HNO}}_{3}$$

In order to assess these particle-phase composition and growth dynamics, we examined upper tropospheric isoprene oxidation in the Fall 2023 Cosmics Leaving OUtdoor Droplets (CLOUD) measurement campaign.

Lopez et al.^14^ provides a theoretical description and test case of a growth-activity phase space and framework called the diagonal volatility basis set (dVBS). The dVBS has important features, including the appearance of a diagnostic infeasible region where species cannot appear when undergoing steady-state, condensational growth alone. Full application of the dVBS requires four elements: an accurate measurement of all potentially condensable vapors, an accurate measurement of speciated particle composition (mass fractions of all species), an accurate measurement of particle growth rates, and an accurate estimation of intrinsic compound volatility. Here, we use dVBS comprehensive gas- and particle-phase measurements at CLOUD in varied combinations to understand the drivers of particle growth and the importance of particle-phase processing during isoprene oxidation and growth in the upper troposphere.

## 2 Materials and Methods

### 2.1 CLOUD Experiments

The CLOUD chamber has been described extensively in previous publications.^15–18^ The specific isoprene oxidation experiments discussed and analyzed here are presented in Shen et al.^8^ In brief, the chamber is a 26.1 m^3^ stainless steel cylinder in a thermal housing capable of temperatures ranging from 213 to 373 K with a high precision gas injection system for trace gases including isoprene, SO_2_, O_3_, NO, and NO_2_. Synthetic air containing nitrogen and oxygen is generated from cryogenic liquids, and the chamber is operated as a continuously stirred tank reactor. Radicals are generated by various chamber lights. Instrument sampling occurs from ports and sampling probes in the midplane of the chamber along the centerline flow. Experiments were conducted at 223 and 243 K to emulate upper tropospheric conditions. During these measurements, oxidation of isoprene produced isoprene-oxygenated organic molecules (IP-OOM) with a sufficiently low volatility to nucleate and grow new particles. Table 1 shows the range of experimental conditions for the full set of experiments analyzed in Section 3.4 and the specific conditions for run 2620.14 analyzed in detail in Sections 3.1, 3.2, and 3.3.

**Table 1:** Conditions across all Experiments and Specific Conditions for Run 2620.14 Discussed in Sections 3.1, 3.2, and 3.3

parameter (units)	range of conditions	run 2620.14 conditions
experiment date	Sep 2023–Nov 2023	October 15, 2023
temperature (K)	223 to 243	223
isoprene (cm^–3^)	1.4 × 10^9^ to 4.2 × 10^10^	1.4 × 10^10^
H_2_SO_4_ (cm^–3^)	<2 × 10^4^ to 7.6 × 10^6^	<2 × 10^4^
NO (cm^–3^)	<2 × 10^8^ to 6.8 × 10^9^	1.5 × 10^9^
NO_2_ (cm^–3^)	1.1 × 10^9^ to 2.4 × 10^10^	1.9 × 10^10^
GR_8–20_ (nm h^–1^)	0 to 34.6	21.4

### 2.2 Instrumentation

Data from a broad range of instrumentation are used to constrain the total “state” of the CLOUD chamber in terms of concentrations, compositions, size, and charge, at high time resolution. Gas-phase concentrations including sulfuric acid, volatile organic compound (VOC) precursors, and oxidized products reported are from the NO_3_^–^ long time-of-flight, chemical ionization mass spectrometer (NO_3_^–^-LToF-CIMS or NO_3_^–^-CIMS, Aerodyne Research, Inc. and Tofwerk AG),^19,20^ the Br^–^ Multischeme chemical IONization inlet-2 mass spectrometer (Br^–^-MION2-LToF-CIMS or Br^–^-MION2, Karsa Ltd.),^21^ and the NH_4_^+^ chemical ionization mass spectrometer (NH_4_^+^-CIMS)^22^ based on the proton transfer reaction mass spectrometer (PTR3) platform.^23^ Using this wide set of mass spectrometers allows for high sensitivity across a wide range of compound classes. In particular, higher volatility, less-oxidized compounds are preferentially detected in the NH_4_^+^-CIMS, while more highly oxidized species are observed in the Br^–^-MION2 with even higher oxygen-to-carbon ratios being most easily detected in the NO_3_^–^-CIMS.^24^ Furthermore, different organic functionalities can be detected preferentially by each reagent ion. Isotopically labeled nitric acid was used in the NO_3_^–^-CIMS to improve detection and unambiguous identification of organonitrate species. Gas-phase sampling lines are chilled to chamber temperature to reduce losses due to thermal fragmentation or other decomposition.

Given the broad set of species observed by these mass spectrometers and the substantial measurement overlap, gas-phase data reported and analyzed here are assessed according to the same metrics as Shen et al.^8^ That study and this one use gas-phase measurements of isoprene oxidation products and acids, including sulfuric acid. Using the definition from Shen et al.,^8^ IP-OOM compounds have the formula C_*i*_H_*j*_O_*k*_N_*l*,_ where *i*, *j* ≥ 4 and *l* = 0 – 2. The presence of nitrogen is associated with a nitrate functional group (−ONO_2_). Previous work has shown that hydroxy (−OH) and nitrate functionalities have similar volatility reductions,^25^ as such the requirement (*k* – 2*l*) ≥ 3 is also applied to give an effective oxygen number of 3 or greater. IP_0N_ refers to IP-OOM with no nitrate functionality, while IP_1N_ and IP_2N_ refer to those with one or two nitrates, respectively. IP_0–2N_ is the sum of IP_0N_, IP_1N_, and IP_2N_, and it is the total IP-OOM measurement. Where gas-phase measurements of individual IP_0–2N_ species overlap in the mass spectrometer data, the algorithm described in Shen et al.^8^ applies. Essentially, during instrument cross-comparison of the same high resolution fit elemental formula, the instrument with the highest concentration measurement is used if the time series behavior of the species across both instruments is correlated with a correlation coefficient of 0.5 or higher. The higher concentration measurement is used because the higher reported signal indicates increased sensitivity to a given species, closer to the kinetic limit used for calibration. Where multiple instruments measure ions with the same elemental formula but the correlation coefficient is less than 0.5, both measurements are used and are considered different isomers. This screening means that the majority of compounds studied here are measured by the Br^–^-MION2 and NO_3_^–^-CIMS, with few species detected by the NH_4_^+^-CIMS. Gas-phase data from the filter sampling chemical ionization mass spectrometer (FIGAERO–CIMS) is not included in this algorithmic analysis since no explicit calibration for sensitivity at the kinetic limit is available and the low-pressure iodide CIMS has a higher LOD, and substantial detection overlap with atmospheric-pressure Br-MION2 measurements.

Sulfuric acid calibration systems^26^ were used to calibrate both the NO_3_^–^-CIMS and the Br^–^-MION2, providing calibration factors of 1 × 10^10^ molec. cm^–3^ per normalized counts per second (ncps) and 1.9 × 10^10^ cm^–3^ per ncps, respectively.^8^ This sensitivity was applied to all of the observed species in each instrument. Reported gas-phase concentrations for both instruments represent a lower bound (within calibration uncertainty) because we assume that sulfuric acid reacts with the reagent ion at the kinetic limit. Humidity-dependent calibration with hexanone is used for the NH_4_^+^-CIMS for all organics under consideration here, and hexanone is detected at the kinetic limit under dry conditions. Sulfuric acid calibration factors vary by nearly a factor of 2, so gas-phase concentrations are reported with representative error bars showing this factor of 2 uncertainty. This applies for all species above the instrumental LOD, with species below the nominal limit of detection (LOD) being more uncertain. The nominal LOD for the Br^–^-MION2 is approximately 10^–4^ μg m^–3^ (3 × 10^5^ cm^–3^), while the NO_3_^–^-CIMS has a lower LOD of 10^–5^ μg m^–3^ (3 × 10^4^ cm^–3^). The nominal LOD of the NH_4_^+^-CIMS is approximately 5 × 10^–4^ μg m^–3^ (10^6^ cm^–3^). No compounds below the nominal NH_4_^+^-CIMS LOD are detected here. However, many gas-phase species below the nominal LOD in Br^–^-MION2 and NO_3_^–^-CIMS are detected. Their collective behavior may be informative in this analysis. So, we report gas-phase measurements below 10^–4^ μg m^–3^ given a clear time series response to chamber conditions based on a Pearson correlation coefficient greater than 0.5 when compared to an appropriate high concentration oxidation tracer. Additional details on Pearson correlation coefficient analysis are provided in the Supporting Information.

Growth rates are derived from particle size distribution measurements from the Neutral cluster and Air Ion Spectrometer (NAIS).^27^ Growth rates from 8 nm up to 20 nm are calculated using the 50% appearance time method.^28^ Additional details on instrument operation and data analysis can be found in a prior publication.^8^

Particle composition is analyzed using the Filter Inlet for Gases and AEROsols coupled to an iodide mode chemical ionization mass spectrometer (I^–^-FIGAERO-LToF-CIMS or FIGAERO, Aerodyne Research, Inc.).^29^ This instrument collects particles onto a PTFE filter through an insulated sampling line over 30 to 60 min, and particles are desorbed from the filter using heated nitrogen ramped from room temperature to approximately 180 °C. This measures chemically speciated particle composition. To reduce the influence of humidity on FIGAERO measurements,^30^ reagent ion flow was humidified using a bubbler to >80% RH. FIGAERO data were analyzed in Igor Pro 9 (Wavemetrics, Inc.) using the Tofware software package (Tofware v3.3.0, Aerodyne Research Inc.) for high resolution mass spectrum peak fitting. Thermogram areas were calculated based on area under best fit Gaussian peaks with constant baseline subtraction and are analyzed assuming a constant sensitivity across all compounds. All particle-phase signals presented have clear desorption behavior above the subtracted signal baseline. Particle mass fraction was calculated by dividing the individual thermogram area over sum total thermogram area, which includes the area of thermograms for unidentified peaks in the mass spectrum with desorption behavior.

### 2.3 Diagonal Volatility Basis Set

The underlying theoretical and graphical framework used here is described in detail in Lopez et al.^14^ To summarize, suspended phase activity (*a* ^s^) and gas-phase activity (*a* ^v^) are equal at equilibrium by definition. Given this, we can construct an equilibrium activity space with the gas-phase mass concentration (*c* ^v^) and particle-phase mass fraction (*w* ^s^) as proxies. A true representation of the activity space would modify these values by the appropriate activity; here, we assume that the mixture of similar products from a common precursor (isoprene) is a sufficiently ideal mixture to use unit activity coefficient, with mass fraction giving the activity. In this equilibrium space, the location of each compound is further constrained by intrinsic volatility, with the decadal volatility bins^31,32^ appearing as diagonal bands in this space. Thus, we call this space, the dVBS. For volatility (*c*°[*T*]) measured in μg m^–3^, the volatility bins span orders of magnitude in log­(*c*°[*T*]) space from volatile organic compounds (VOC, log­(*c*°[*T*]) > 6.5) into intermediate (IVOC, log­(*c*°[*T*]) = 2.5 to 6.5), semivolatile (SVOC, log­(*c*°[*T*]) = −0.5 to 2.5), low (LVOC, log­(*c*°[*T*]) = −4.5 to −0.5), extremely low (ELVOC, log­(*c*°[*T*])) = −8.5 to −4.5), and ultra-low (ULVOC, log­(*c*°[*T*]) < −8.5) volatility bins.

In this study, volatility at 300 K for most compounds is calculated based on carbon, oxygen, and nitrogen numbers (*n*_C_, *n*_O_, and *n*_N_, respectively) according to the elemental parametrization used in Stolzenburg et al.^33^ For a small subset of IP-OOM for which we have a reasonable sense of their structure based on molecular mechanisms,^7,34^ the SIMPOL group contribution model is used to assess their volatility.^35^ These volatility estimates are then adjusted for the appropriate measurement temperature in the CLOUD chamber using a previously described method based on the enthalpy of vaporization,^36^ though the updated parameters from Stolzenburg et al.^33^ are used here. Further discussion can be found in the Supporting Information.

Particles rarely float about at a gentle equilibrium in the atmosphere. Typically, they grow. For steady-state growth, the flux of material to the particle is totally constrained to maintain the growth of each “shell” of the material. In addition, the available condensing vapors are kinetically limited to a specific maximum speed. As such, an excess vapor concentration (*c*_p_^xs^) is defined as the concentration of a single vapor condensing at the kinetic limit that would provide the total mass flux to the particles necessary to maintain the constant growth rate. For typical organics in the atmosphere, (ρ_p_ = 1.4 g cm^–3^, MW_avg_ = 300 g mol^–1^), the growth rate (*R*_p_^gr^) is related to the excess vapor concentration as follows in eq 1 here (reproduced from Lopez et al.^14^):

$$R_{p}^{\mathrm{gr}}=184\frac{\mathrm{nm}\;h^{-1}}{\mu g\;{\mathrm{cm}}^{-3}}c_{p}^{\mathrm{xs}}$$ 1

This defines the right y-intercept in the dVBS space, where a given growth rate dictates a required total excess concentration of vapor as a minimum requirement for the observed growth. For multicomponent systems, the minimum required vapor phase concentration for each component *i* necessary to achieve a particular particle-phase activity (*c*_*i*,min_^v^) is also determined by the kinetically limited case. For steady-state particle growth, a diagonal “kinetic condensation” line is driven across the equilibrium space with the formula shown in eq 2. This provides the lower bound on gas-phase concentration for a given particle mass fraction if nonreactive condensation controls steady-state growth.

$$c_{i,\min}^{v}=c_{p}^{\mathrm{xs}}a_{i}^{s}$$ 2

To aid interpretation of the dVBS growth space, a labeled diagram of the space is shown in the Supporting Information (Figure S1). The *x*–*y* coordinate of each point indicates its particle-phase mass fraction and gas-phase concentration, respectively. Note that the dVBS figures are built vertically on the right axis, where gas-phase mass concentrations in μg m^–3^ are reported. Concentrations on the left axis in molec. cm^–3^ are estimated based on an average molecular weight of 300 g mol^–1^ to guide readers more familiar with these units. Similarly, the growth rate axis contains implicit density and molecular weight assumptions, as shown in eq 1. Figure S1 indicates how to interpret the total growth rate and individual component contributions to growth.

For complex atmospheric mixtures, sufficiently low-volatility species fall on the kinetic condensation line as they condense kinetically to the particle, while higher volatility species largely appear as they would be at equilibrium at the position defined by their volatility, with only small increases in gas-phase concentrations out of equilibrium to maintain growth. This diagonal line also defines an infeasible region where certain pairings of high particle mass fraction and low vapor-phase concentration exist and cannot occur if nonreactive condensation dominates steady-state particle growth. As such, this region can probe growth-relevant particle processes such as hydrolysis, oligomerization, and reactive uptake. Similarly, higher volatility species that appear outside of their equilibrium positions may indicate inhibited uptake or condensed-phase processes.

The dVBS can be applied to nucleation and growth experiments like those at CLOUD because steady-state gas-phase concentrations and constant growth rates can be maintained. For more dynamic experiments and measurements, the particles integrate over their lifetime, and low-volatility species which are formed early in the process may have a high particle mass fraction without correspondingly high gas-phase concentrations. The dVBS analysis presented here is unlikely to apply to most field studies due to the mixing of multiple air masses, shifting condensation sinks, and unstable growth rates.

For isoprene oxidation, condensed phase processing has been observed in field and laboratory studies, but the contribution during initial growth is unknown. In the case of particle-phase oligomerization reactions,^6^ such a process would result in the formation of particle-phase dimers with a higher concentration in the particle-phase than expected. These dimers would be driven into the infeasible region, while particle-phase concentrations of the reagent monomers would be systematically reduced. Similarly, processes like organonitrate hydrolysis which transforms organonitrate functional groups into hydroxy functionalities^11^ would enhance a non-nitrate IP-OOM in the particle-phase while depleting the nitrate form. This study will probe the contribution of these processes to the growth of small isoprene particles. The influence of other types of particle-phase processes in this space is discussed in the study by Lopez et al.^14^

## 3 Results and Discussion

The dVBS provides a highly constrained phase space, where the interplay of volatility, growth, and gas and particle composition allows for a wide range of predictions dependent on the available or chosen measurements. Here, we will examine in detail an isoprene oxidation, nucleation, and growth experiment from CLOUD (Run 2620.14) whose conditions are summarized in Table 1. This experiment was conducted with background sulfuric acid in the chamber and in the presence of NO_X_ at 223 K. First, given gas-phase composition, volatility, and growth rate data alone, we calculate the expected particle-phase composition due to nonreactive condensation. Next, we compare this predicted particle-phase composition to that measured by the FIGAERO–CIMS and assess the influence of reactive particle-phase processes, uptake inhibition, and other observed deviations from nonreactive condensation. Then, we briefly assess combined gas and particle measurements’ prediction of growth rates. Finally, since many experiments failed to generate sufficient aerosol mass for analysis in FIGAERO–CIMS, we use the dVBS to predict growth rates for a broad set of isoprene oxidation experiments at CLOUD and assess the major drivers of growth across all these experimental conditions.

### 3.1 Measured Gas-Phase and Calculated Particle-Phase Composition

In Figure 1, we use measurements of the gas-phase composition and growth rate along with estimated volatility to predict particle-phase composition from an isoprene oxidation experiment during steady-state growth. We assume that measured gas-phase concentrations are accurate and comprehensive within a factor 2 uncertainty in concentration. Nominal instrumental LODs in the gas and particle phase are shown as dashed lines in Figure 1. For compounds below a nominal gas-phase LOD of 10^–4^ μg m^–3^, their measurement uncertainty is even higher than this nominal factor 2. However, their collective behavior is still interesting. So, they are reported here if their Pearson correlation coefficient with key gas-phase oxidation tracers is greater than 0.5 as discussed in the Supporting Information. Gas-phase concentrations are at an approximate steady state during this growth event, and the measured growth rate is reasonably stable, such that the dVBS can be applied.

**Figure 1:**
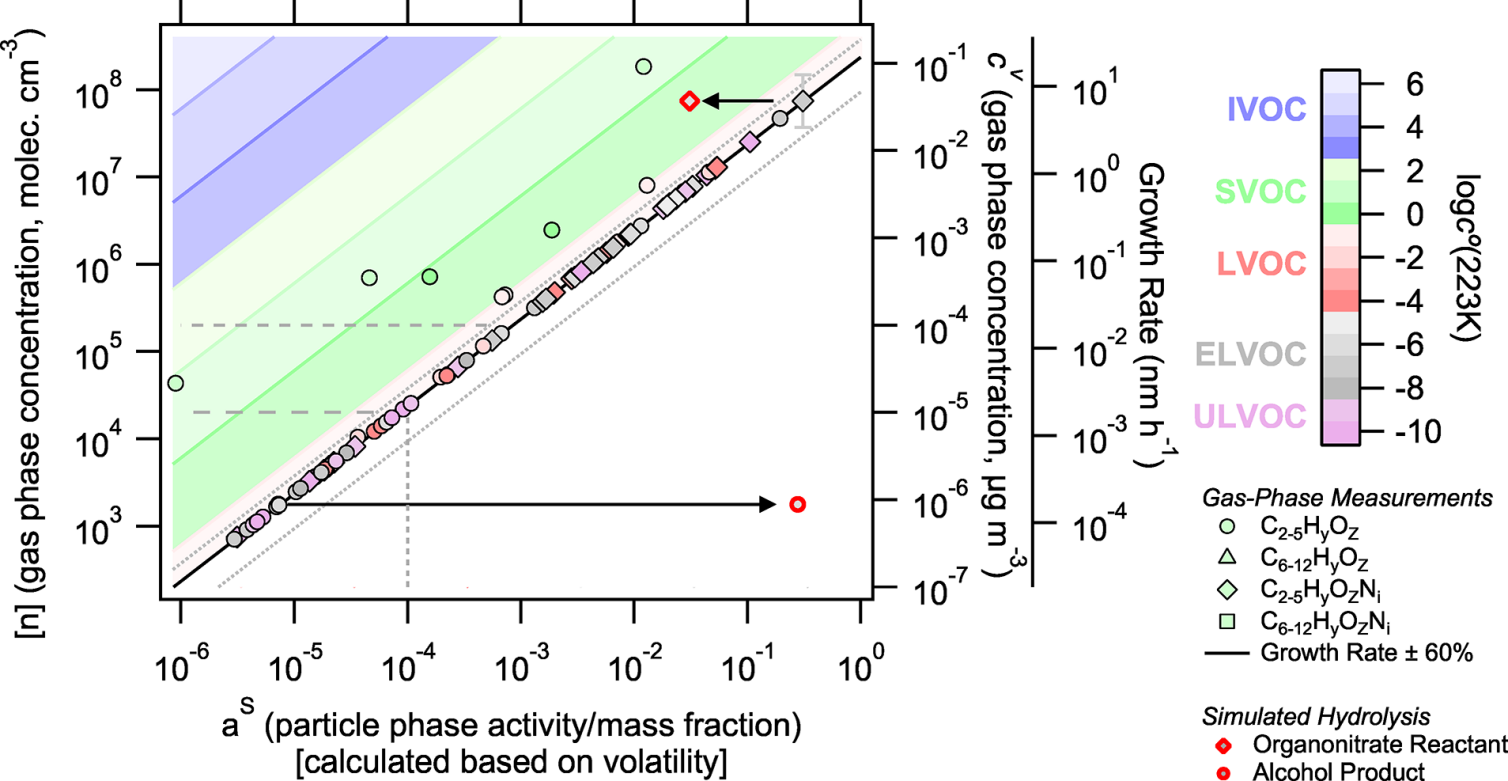
Predicted particle-phase composition (*x*-axis) according to the dVBS based on measured gas-phase concentrations (left and right *y*-axis) and growth rate (right y-intercept) and estimated volatility for an isoprene oxidation experiment at 223 K. Symbol shapes indicate carbon and nitrogen contents, and symbol color reflects estimated compound volatility. A representative vertical error bar is shown on the most rightward point, representing the factor 2 uncertainty in gas-phase measurement. Growth rate ± 60% is shown in solid and dashed diagonal lines. Dashed horizontal lines show the nominal LODs of gas-phase instruments (Br^–^-MION2 LOD at *c* ^v^ = 10^–4^ μg m^–3^ and NO_3_^–^-CIMS LOD at *c* ^v^ = 10^–5^ μg m^–3^). Compounds measured below *c* ^v^ = 10^–4^ μg m^–3^ are reported only if they are correlated with key oxidation tracers (*R* > 0.5). The dashed vertical line at *a* ^s^ = 10^–4^ shows the estimated LOD of the particle-phase composition measurements. Red-bordered symbols illustrate the simulated influence of organonitrate hydrolysis in the particle-phase. We simulate the case where 90% of the organonitrate reactant is consumed and the alcohol product would appear at its equilibrium volatility position in the “infeasible region”.

In these dVBS figures, molecules without nitrogen and with *n*_C_ ≤ 5 are plotted as circles, molecules without nitrogen and *n*_C_ > 5 are plotted as triangles, molecules with nitrogen (*n*_N_ > 0) and *n*_C_ ≤ 5 are plotted as diamonds, and molecules with *n*_N_ > 0 and *n*_C_ > 5 are plotted as squares. We shall refer to species with *n*_C_ ≤ 5 as “monomers”, because the precursor isoprene has *n*_C_ = 5, and species with *n*_C_ > 5 as “dimers”. We shall refer to species with nitrogen as “nitrates” assuming that RO_2_ + NO reactions are the major nitrogen incorporation pathway.^7,8,34^ Note that the right intercept of the growth rate line is defined by the measured 21.4 nm h^–1^ steady-state growth rate.

Based on vapor and growth rate measurements alone, the dVBS predicts based on nonreactive condensation that particle growth and composition would be dominated by low-volatility species crowded onto the kinetic limiting line. A mononitrate species (C_5_H_11_O_4_NO_3_) would make up over 30% of the particle mass in this system. A more conventional oxidized organic compound (C_5_H_12_O_6_) would be the most substantial non-nitrate contributor in this calculation, making up 18% of the particle mass. There are few semivolatile species, with only one vapor (C_5_H_10_O_3_) measured at a level (9.1 × 10^–2^ μg m^–3^ or 1.8 × 10^8^ cm^–3^) that would nearly double the growth rate if it were low enough volatility to condense kinetically. This is the highest concentration of IP-OOM, but it is an SVOC that is predicted to comprise about 1% of the particle mass. This aligns reasonably well with the chemistry observed above the Amazon, where C_5_H_11_O_4_NO_3_ was a substantial vapor during flight through a nucleating plume.^7^ Furthermore, C_5_H_12_O_6_ and similar species were observed in prior experiments and this experimental set to be key species in particle growth from isoprene oxidation.^8^

The general composition of gases predicts growth controlled by a small set of low-volatility species at these cold temperatures. ELVOCs would make up a majority of the particle mass at 63% and ULVOCs would make up an additional 19%. LVOCs would make up 15% of the particle mass. Of the 181 IP-OOM measured, only 40 species would have *a* ^s^ ≥ 0.001 and make up 99% of the particle mass. C_5_ monomers are expected to dominate this set, making up 82% of the particle mass, while C_6–12_ dimers would make up another 3% and fragmentation products would cover the balance. For this system, a nearly complete accounting of the particle composition is expected by measuring species with gas-phase concentrations greater than the nominal gas-phase instrumental LOD of 10^–4^ μg m^–3^ (3 × 10^5^ cm^–3^). All compounds above this nominal gas-phase instrumental LOD are expected to make up more than 0.1% of the particle-phase mass fraction and be above the expected LOD of the FIGAERO particle composition measurement (*a* ^s^ = 10^–4^).

The red symbols in Figure 1 show how particle-phase processing could appear in this space. If an abundant organonitrate species undergoes hydrolysis in the particle-phase, it would shift left as it was consumed by hydrolysis (by 90% in this example). If the product of this hydrolysis were a low-volatility species with no substantial gas-phase source, it would appear in the particle-phase with a particle-phase mass fraction approximately equal to the initial mass fraction of the organonitrate and a gas-phase concentration dictated by its volatility, an obvious point deep in the infeasible region. In this example, the nitrate reactant is an ELVOC that would have condensed with unit efficiency without hydrolysis. Therefore, hydrolysis would not change the growth rate by much. If the SVOC, C_5_H_10_O_3_, at 1% particle fraction and 1.8 × 10^8^ cm^–3^ gas-phase concentration were to undergo reactive uptake with a near unity uptake coefficient to form a low-volatility product, that product would appear in the infeasible region and increase the growth rate by approximately 16 nm h^–1^.

### 3.2 FIGAERO Particle Composition

After predicting the particle-phase composition based on growth rate, gas composition, and volatility, we assess the measured particle composition. In this study, the only measurement of particle composition was the FIGAERO–CIMS. Figure 2 reprises the dVBS from Figure 1, but with particle-phase composition measured by FIGAERO. It is noteworthy that we fail to capture all the species observed by the gas-phase mass spectrometers in our particle measurement. This is expected, given the varying sensitivity of reagent ions to specific analytes,^24^ with more highly oxidized species often undetected in the I^–^-CIMS. We plot gas-phase species with no measured particle signal but a calculated particle-phase mass fraction greater than 10^–3^ to the left of the main region of Figure 2 marked with a gray *x*-axis indicating carbon number. The dVBS predicts that these unmeasured species would comprise about 30% of the particle mass, and so, our FIGAERO measurements capture about 70% of the particle mass composition. Many undetected species are organonitrates, and systemic sensitivity considerations will be discussed later. For now, the infeasible region draws the eye toward dimer triangles clustered near the bottom of the plot.

**Figure 2:**
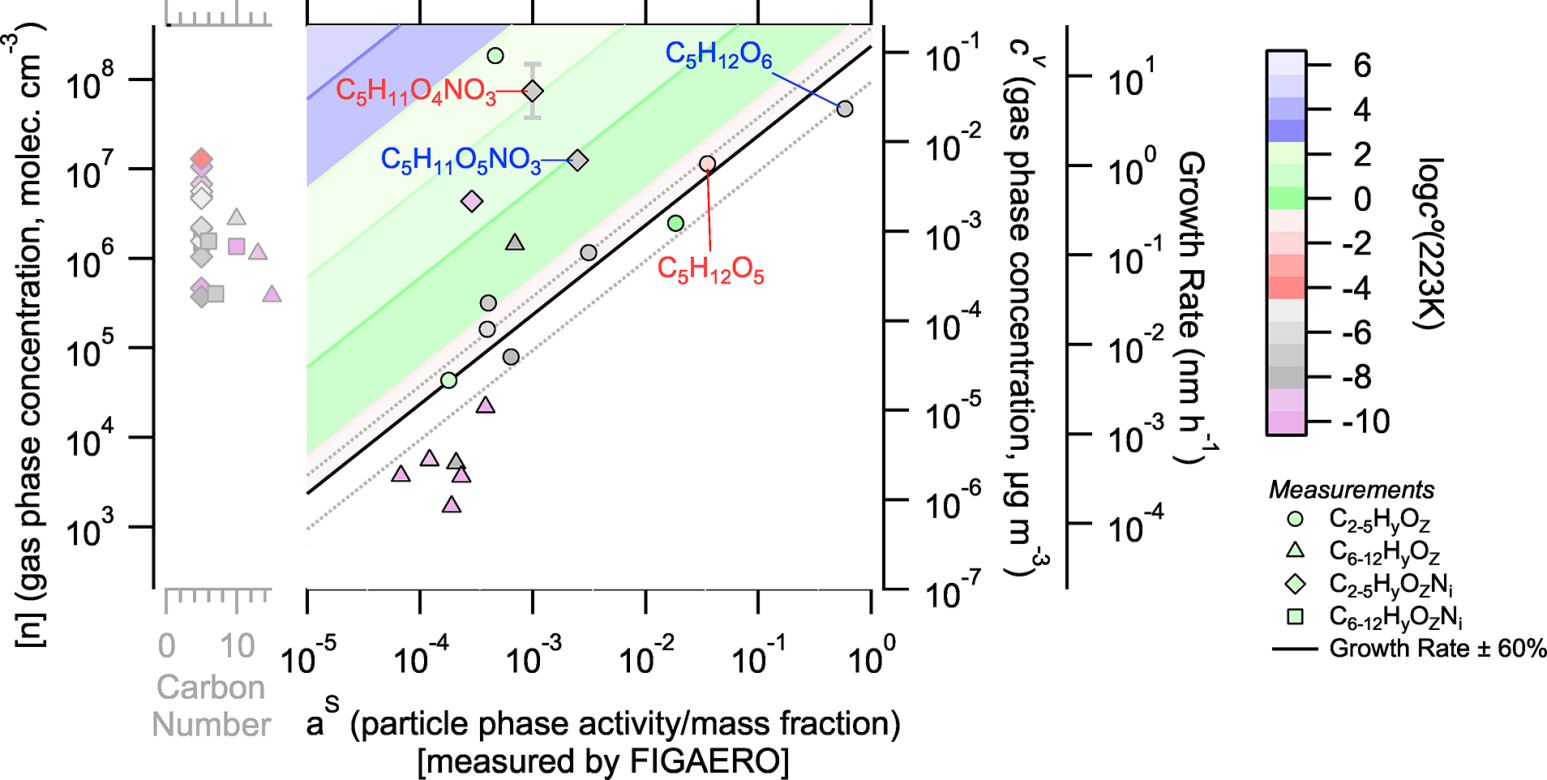
dVBS for the isoprene oxidation experiment at 223 K with gas concentration and growth rates from the same sources as Figure 1, now incorporating particle-phase composition data from the FIGAERO. FIGAERO data here assume uniform sensitivity across observed compounds. The representative vertical error bar on C_5_H_11_O_4_NO_3_ again represents a factor of 2 uncertainty in measured gas-phase concentrations. Those species predicted to have *a* ^s^ > 10^–3^ but not observed by FIGAERO are included in the gray axis on the left of the plot. In this case, approximately 70% of the particle composition is accounted for by the FIGAERO measurement. Blue and red labels denote four species that make up potential hydrolysis organonitrate reactant and alcohol product pairs.

#### 3.2.1 Enhancement of Dimers in Small Particles Drives a Shift in Observed Composition

A group of dimers stands out in the infeasible region of Figure 2, albeit at a very low signal with *a* ^s^ < 0.001 and [*n*] < 10^4^ cm^–3^. None contain nitrogen. Dimers in the infeasible region may be formed in the particle-phase by oligomerization reactions, driving their relative enhancement compared to their gas-phase concentrations. Dimers may also dominate small particles, as discussed later. However, dimer gas-phase concentrations here are below the nominal LOD of the gas-phase mass spectrometers and are highly uncertain. Further, these dimers make a very small contribution to particle mass and collectively comprise roughly 1% of the particle mass. This cluster likely shows the limits of detection in both gas- and particle-phase measurements. In this case, particle mass fractions and gas-phase concentrations are poorly constrained for the dimers.

However, initial nucleating clusters are dominated by dimer–dimer interactions such that dimers dominate the composition of the smallest particles during α-pinene oxidation.^37^ Thus, monomer contributions are reduced by the Kelvin effect during initial growth.^38,39^ Dimers may be enhanced in 4–5 nm particles, which would displace them slightly into the infeasible region. However, a dynamic VBS model^32^ shows a single nucleation species would be increased by approximately 10^–4^ in 20 nm particles (Figure S2 in the Supporting Information). This total increase would have to be spread across all of the enhanced dimers and would not account for their total enhancement. Furthermore, Shen et al.^8^ finds the monomers contribute substantially to nucleating clusters during isoprene oxidation, reducing such dimer enhancements. Microphysical explanations cannot account for the overall enhancements shown here; therefore, uncertainty in instrumental measurement is more reasonable.

Other than the cluster of dimers near the detection limit, no other dimers appear in the infeasible region of Figure 2. We conclude that there is no evidence for significant particle-phase oligomerization of isoprene oxidation products. Even if the dimer enhancements are real, dimers make up a sparingly small fraction of the total particle mass and are dwarfed by condensation of a small set of monomers controlling overall particle growth.

#### 3.2.2 Organonitrate Sensitivity

Figure 2 appears to be consistent with organonitrate hydrolysis. Specifically, several nitrates have lower than expected particle-phase abundance, and their corresponding hydrolysis products are enhanced. For example, C_5_H_12_O_6_ is to the right of the kinetic limiting line, while its potential source from hydrolysis, the organonitrate C_5_H_11_O_5_NO_3_, appears to the left of the limiting line, consistent with depletion from reactive uptake. C_5_H_12_O_6_ also appears in the infeasible region; however, this species also has a substantial gas-phase concentration, so any hydrolysis would augment an already high particle mass fraction. Similarly, C_5_H_12_O_5_ and C_5_H_11_O_4_NO_3_ form a hydrolysis pair that shows substantial displacement of the nitrate away from the kinetic limiting line and enhancement of the non-nitrate. While these pairs are qualitatively consistent with organonitrate hydrolysis, the quantitative mass balance is not satisfied. The apparent particle-phase nitrate consumption does not explain apparent product formation.

To assess the potential hydrolysis mass balance, it is important to understand where the hydrolysis products would appear in this space if they were formed exclusively in the particles. First, the nitrate signals are underrepresented in the particles. Various volatility estimation methods^31,33,35^ agree that these nitrates and their hydrolysis products should be low enough volatility to condense kinetically (or within 1 decade of the condensation line) at 223 K for growth rates within the reported range. Further, Shen et al.^8^ finds that IP_1–2N_ species must condense near kinetically to explain growth during these isoprene oxidation experiments. Instead, the nitrate signals are displaced by 1–2 orders of magnitude to the left of the condensation line. This could mean that 90–99% of these were consumed by hydrolysis in the particle (shifting the *x*-axis value left), but the resulting alcohol products also have a low volatility. If this were the only source of the hydrolysis products, their gas-phase signal would be at most their equilibrium value, which in each case is far below the condensation line. In fact, their gas-phase signal would be lower than equilibrium due to wall losses (unless the aerosol condensation sink was far greater than the wall loss sink). This is precisely why the products would appear deep in the infeasible region, with a high particle-phase but very low gas-phase signal. Instead, these species appear close to where they should if condensation was the main source in the particles; they have high gas-phase concentrations, low volatility, and high particle-phase fractions. C_5_H_12_O_6_ does “tip-toe” into the infeasible region, and given this log–log scale, this could indicate that half or even somewhat more of it does come from hydrolysis, but it is not overwhelming evidence. Rather, we turn to instrumental explanations again.

First, it is possible that the apparent reduction in the particle-phase fraction for these organonitrate species is a case of mistaken isomer identity. If the gas- and particle-phase mass spectrometer measurements were preferentially detecting different isomers of these nitrates, their behavior in dVBS could be dramatically different. Further, these species may not be nitrates but peroxynitrates.^40^ Peroxynirates would decompose rapidly during thermal desorption and not be directly detected.^41^ This decomposition is primarily driven toward the peroxy radical (ROO^·^)^42^ which could form several products including the alcohol product of RONO_2_ hydrolysis. However, we cannot meaningfully distinguish between such isomer detections. There is no clear evidence of early thermal decomposition in the organonitrate thermograms or detection of products in the particle phase (other than the organonitrate hydrolysis product). Thus, we assume in our further analysis that these species are organonitrates and the same isomer across mass spectrometers.

In Figure 2, we inferred the particle mass fraction based on the I^–^ FIGAERO signal, assuming a constant sensitivity. This is not correct as I^–^ chemical ionization is not universally efficient.^43^ However, although we only have I^–^ CIMS measurements for the particle-phase, we have a suite of gas-phase measurements, as discussed above. Intercomparison of calibrated NO_3_^–^-CIMS and Br-MION2 gas-phase measurements and I^–^-CIMS gas-phase signal for these shared non-nitrate/organonitrate species shows that the sensitivity of the I^–^-CIMS to these organonitrates is reduced by 1 to 2 orders of magnitude (Figure S3 in the Supporting Information). In CIMS, the highest concentration “wins” with a maximum possible detection efficiency of 1.0, defined by the collision limit of the reagent ion with analyte molecules. Calibration of the NO_3_^–^-CIMS and Br-MION2 using sulfuric acid and the application of this collision-limited calibration factor across all species quantifies a lower bound on organonitrate concentrations across the instrument ensemble. So we conclude that the organonitrate detection efficiency reduced by a factor to 10 to 100 for the I^–^-CIMS measurements. Both gas- and particle-phase measurements in the FIGAERO are carried out in the same ion–molecule reactor, with humidified reagent gas, so we expect the detection efficiency for each species to be identical for each phase. Based on this, we adjust the raw I^–^ signals for organonitrates by multiplying their integrated desorption area by a factor of 30 (Figure 3) and show the particle composition range given adjustments by factors of 10 and 100. This also explains why the cluster of dimers discussed above contains no nitrate dimers; even with similar mass fraction, their signals would just be too low to observe.

**Figure 3:**
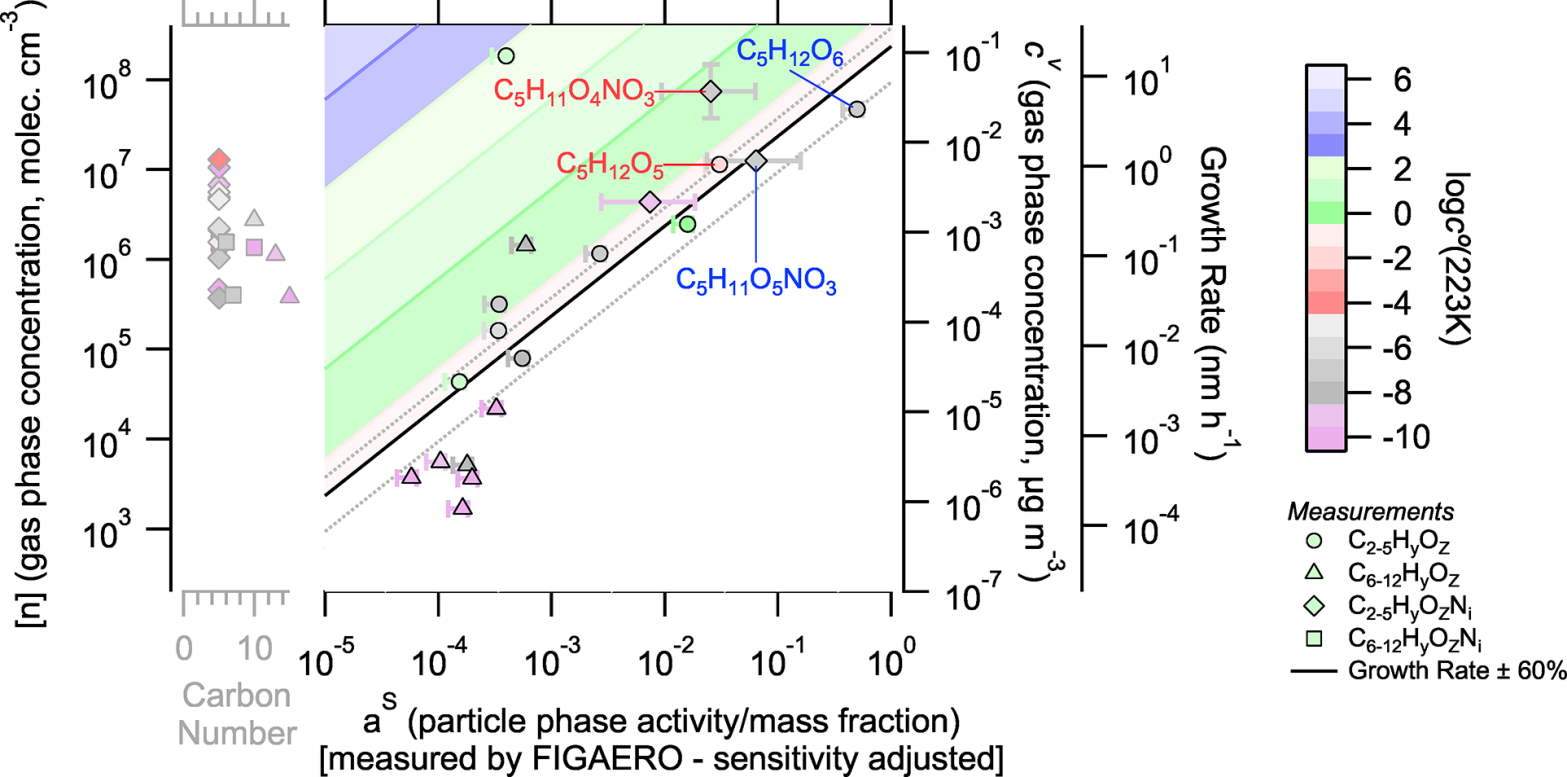
Gas–particle activity space similar to that in Figure 2 adjusted for estimated I^–^-CIMS sensitivity to organonitrate species. Particle-phase organonitrate signals are shifted by a factor of 30. The representative vertical error bar on C_5_H_11_O_4_NO_3_ still represents a factor 2 measurement uncertainty for species above nominal LOD. Horizontal error bars indicate the range of composition shifts if organonitrate signals are instead multiplied by a factor of 10 or 100.

Other than using the gas-phase I^–^ signals to calibrate the particle-phase I^–^ nitrate measurements, the gas-phase and particle-phase measurements are independent. The gas-phase values come from the suite of mass spectrometers (not the FIGAERO), but the particle-phase measurements come from FIGAERO alone. This is important because the dVBS shows that we expect a high degree of correlation in gas- and particle-phase values, and potential crosstalk or other correlated responses (backgrounds, etc.) in the single instrument would be a significant confounding factor. Future explorations and measurements in this space demand additional independent calibration of FIGAERO or other particle composition constraints.

#### 3.2.3 Assessment of Organonitrate Hydrolysis and SVOC Uptake

In Figure 3, we show the dVBS analysis with adjusted organonitrate FIGAERO measurements. Over a range of about 3 orders of magnitude, the combined gas and particle measurements fall along the diagonal condensation line, consistent with nonreactive condensation of effective nonvolatile species. We can now assess organonitrate hydrolysis and the behavior of equilibrium species in the adjusted space.

The first potential hydrolysis pairs (C_5_H_12_O_6_ and C_5_H_11_O_5_NO_3_) are now both near the boundary of the kinetic growth regime at the maximum boundary of growth rate uncertainty. Both species are underestimated by the calculation in Figure 1, but are near enough to the observed growth rate uncertainty. The other hydrolysis pairs labeled here (C_5_H_12_O_5_ and C_5_H_11_O_4_NO_3_) show some minor evidence of hydrolysis. However, the shift in the non-nitrate OOM is still insufficient to capture the observed depletion of the nitrate species and this variation is well within the uncertainty in instrumental sensitivity. The other organonitrate species observed have only minor contributions to particle-phase composition and show minimal consumption. Finally, for that set of organonitrate species which are not observed in the particle-phase, represented in gray on Figures 2 and 3, there is no corresponding transformation product in the particle-phase for any of these species which we fail to measure in the FIGAERO. Under these conditions, organonitrate hydrolysis does not appear to be a relevant process controlling the growth of isoprene-derived nanoparticles. However, under warmer conditions where aerosol water content may be enhanced and the volatility of IP-OOMs is higher, such processes may have some influence.

We measured three equilibrium (SVOC) species in the gas- and particle-phase. The one major semivolatile gas-phase species appears well to the left of condensation line, within the (green) semivolatile region as expected. We failed to detect other SVOC species, which appear in Figure 1 with low expected mass fractions. The species C_5_H_10_O_3_ appear in the particles at a lower mass fraction than expected (displaced left by roughly a factor of 50). Isoprene oxidation products have been observed to form highly viscous particles under some conditions,^44^ and the displacement of C_5_H_10_O_3_ is potentially consistent with high viscosity inhibition of nonreactive condensation and mixing into the particle phase.^45^ Two additional SVOC species were not detected in the FIGAERO data; however, none were predicted to have a particle mass above 10^–4^. If these species were inhibited by the same factor as C_5_H_10_O_3_, the most abundant compound would have a mass fraction near 5 × 10^–6^ and go undetected (Figure S4). Finally, two SVOC species appear above their expected mass fractions in Figure 3, almost exactly on the condensation line. Their peak desorption temperature is higher than expected based on their calculated volatility (Section S6 of the Supporting Information). This is consistent with reactive uptake and subsequent decomposition upon heating.

The detected SVOCs appear to indicate opposite uptake processes and effects, but this may not be contradictory and in any event has little effect on the overall growth rate. Nonreactive SVOC uptake requires fast diffusion to reach particle-phase activity equilibrium, so diffusion limitations inhibit uptake. However, in cases where the reactive diffusive length of the species is very small, highly efficient reactive uptake may not be inhibited by diffusion limitations.^46^ For an SVOC to appear on the condensation line, the reactive uptake coefficient would need to be unity and diffusion restrictions may not limit this (evidently reversible) reactive uptake. This dVBS analysis alone does not provide conclusive evidence of diffusion limitations or rapid reactive uptake for some volatile species. However, it is consistent with particle growth being driven by effectively nonvolatile, nonreactive uptake of low-volatility species with gas-phase activities much greater than 1. These species condense with unit efficiency, regardless of any diffusion limitations.

This first multi-instrumental application of the growth-activity space, developed by Lopez et al.,^14^ shows that the dVBS is effective at connecting quantified gas-phase measurements with FIGAERO particle composition measurements, though future studies should include multiple particle composition measurements. During low-temperature isoprene oxidation, we find that the growth is dominated by the kinetic condensation of low-volatility species. There is no evidence that reactive uptake enhances growth, via either hydrolysis or dimerization, and indeed no evidence for any substantial reactive uptake. As such, modeling of initial particle growth in the atmosphere from isoprene oxidation may not require condensed phase reactions.

### 3.3 Prediction of Growth Rate from Multiphase Measurements

In the prior analyses, the growth rate in this experiment was calculated based on particle size distribution data. However, growth rates can be predicted in the dVBS by gas and particle measurements. For a given growth rate, the position of the condensation line is fixed by the right-intercept at *a* ^s^ = 1 and eq 2. By sweeping the condensation line across the space with increasing growth rate and calculating the logarithmic orthogonal distance of each point from the line, we determined the predicted growth rate from the gas- and particle-phase measurement ensemble by minimizing the sum of logarithmic orthogonal distances. This distance sum is used as the cost function rather than *x* or *y* root-mean-squared errors because gas and particle compositions are measured and subject to uncertainty. The log-space distance is used because the linear distance fit is dominated by high abundance species with minimal influence from other points. The logarithmic orthogonal distance sum can be weighted by the volatility of each compound using the gamma function shown in eq 3 to reduce the influence of high volatility species.

$$\gamma =\frac{1}{1+\frac{c_{i}^{{}^{\circ}}(T)}{c_{p}^{\mathrm{xs}}}}$$ 3

In Figure S5, we show the growth rate fit for run 2620.14 using this method, with and without organonitrate adjustment in panels a and b, respectively. Growth rates for the unweighted fits are 13.7 and 14.9 nm h^–1^, while γ-weighted fits show slightly decreased growth rates of 11.3 and 13.1 nm h^–1^ due to the reduced influence of C_5_H_10_O_3_. These predicted growth rates are within the uncertainty of the measured growth rate. Furthermore, the factor 2 uncertainty in gas-phase concentrations causes a similar spread in the predicted growth rate (5.6–32.4 nm h^–1^). The fitted growth rate from varying the organonitrate sensitivity factor adjustment from 10 to 100 ranges from 13.1 to 18.0 nm h^–1^. Together, this indicates nonreactive condensation of the species shown is a strong model within measurement uncertainty. Future studies should employ multiple independent measures of particle composition and growth rates to reduce the uncertainty.

### 3.4 Gas-Phase Growth Prediction and Drivers

Where multiphase measurements are not available, the dVBS can still constrain and predict growth rates using gas-phase measurements and estimated volatility. From this single experiment, growth of isoprene-derived aerosol at the low temperatures of the upper troposphere is limited to a small set of nitrate and non-nitrate IP-OOM. Theoretical and measured aerosol composition is dominated by monomer species. Rather than SVOC driven growth, where high concentrations of equilibrium regime compounds drive the majority of the growth, the growth rate in this experiment is associated with the kinetic condensation of gas-phase species.

Using gas-phase measurements, we can explore the drivers of growth across the full experimental set. As shown in Figure 4, measured growth rates from 8 to 20 nm (GR_8–20_) vary substantially across the full set of isoprene oxidation experiments. A simple growth rate prediction can be made by taking the sum of the gas-phase measurements of IP_0–2N_ and converting this sum to an expected growth rate with eq 1. This total vapor measurement prediction correlates strongly with measured growth rates (*R* ^2^ = 0.9) but overestimates them on average by 80%. This is a lower bound on growth rates predicted using this method, given that vapors are treated as if detected at the kinetic limit.

**Figure 4:**
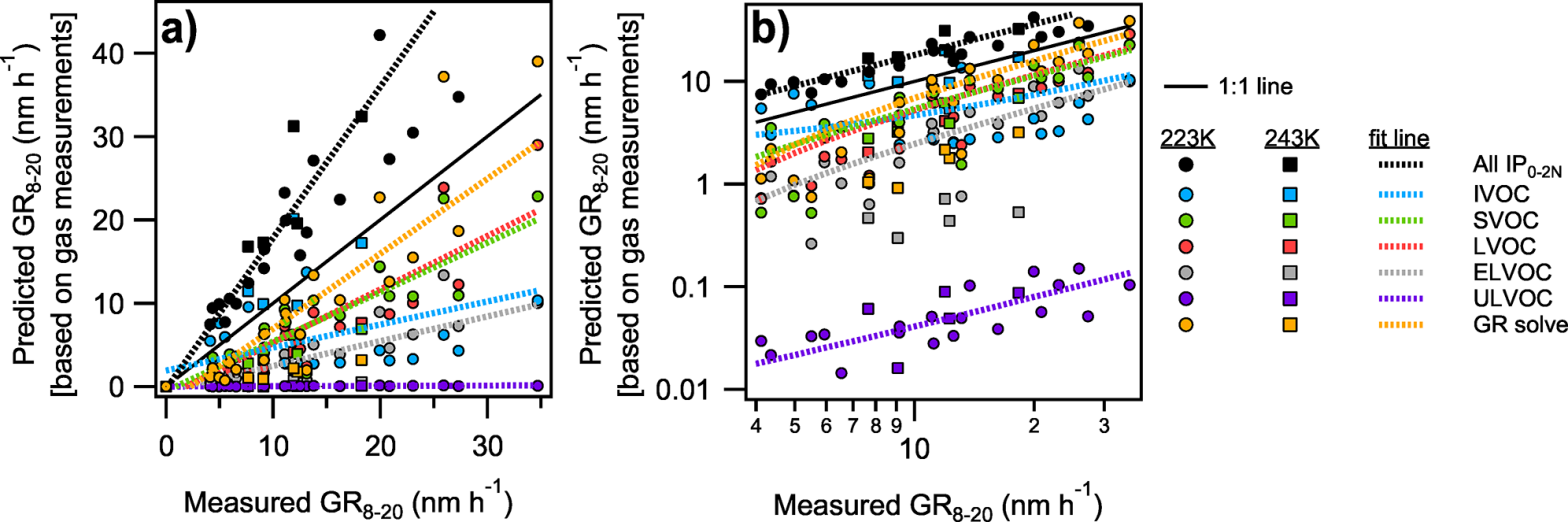
Measured GR_8–20_ for CLOUD16 isoprene growth observations compared to predicted growth rate on (a) linear and (b) log axes. Growth rates are predicted based on total gas-phase measurements, measurements binned by volatility, and the dVBS solution (GR solve). Sulfuric acid is treated as an ULVOC in all cases. Least squares regression generates lines of best fit from data at both 223 K (circles) and 243 K (squares).

To assess how each volatility class contributes to and explains GR_8–20_, compounds are grouped, summed, and converted to an expected growth rate with other species in their temperature-adjusted VBS volatility bin. Sulfuric acid condenses kinetically and is treated as an ULVOC. ULVOC species contribute minimally to growth given their low concentrations. While they are reasonably correlated to growth (*R* ^2^ = 0.7), ULVOCs explain <1% of measured growth. ELVOCs have similar correlation to growth, but only explain 30% of measured growth on average. Conversely, IVOC species have a substantial gas-phase concentration but do not correlate strongly with measured GR_8–20_ (*R* ^2^ = 0.3) due to their high volatility. SVOC and LVOC estimated growth rates are both well correlated with measured growth rates (*R* ^2^ = 0.8 in both cases), but explain 60–65% of measured GR_8–20_ on average. We see high correlation between all VOC volatility classes and growth rate because the oxidation products are highly correlated in these experiments (so IVOC and ULVOC concentrations are highly correlated, for example). Correlation alone is thus not sufficient to test for consistency.

The dVBS provides a quantitative comparison of the vapor concentrations and aerosol growth. Rather than simply adding vapor concentrations, we can calculate the expected growth rate from the volatility basis set in a recursive manner, similar to classic organic aerosol calculations in VBS analysis^47^ and described in Lopez et al.^14^ Following an initial *R*_p_^gr^ guess, the *c*_p_^xs^ is calculated from eq 1. Then, the expected particle-phase mass fraction (*w*_*i*_^s^) of all measured compounds is assessed and summed according to eq 4. The sum of all particle-phase mass fractions should be 1. If it is not (within some numerical tolerance), multiply *R*_p_^gr^ by *w* ^s^ to generate a new growth rate guess and iterate until this process converges.

$$w^{s}=\sum_{i}w_{i}^{s};w_{i}^{s}=\frac{c_{i}^{v}}{c_{p}^{\mathrm{xs}}}\gamma$$ 4

This method of growth rate prediction is shown in Figure 4 as “GR solve”. This gives a correlation of *R* ^2^ = 0.7 and accounts for 90% of measured growth, on average. Kinetic condensation of IP_0–2N_ (defined here as growth caused by compounds with γ > 0.9) accounts for more than 80% of the growth on average. This is why the condensing species lie along the condensation line (which is positioned based on the observed growth rate) in Figure 3. In general, the growth of small particles during isoprene oxidation is dominated by kinetic condensation of sufficiently low-volatility species.

Growth being tightly constrained by kinetic condensation may influence the field and model assessment of isoprene aerosol growth. For example, current aircraft measurements observe large concentrations of IP-OOM present above certain forests.^7^ Nucleation and growth of isoprene oxidation products have been observed in related experimental and modeling studies.^8,9^ Based on our work, isoprene aerosol growth is well constrained by kinetically condensing species, which includes ULVOCs, ELVOCs, and most or all LVOCs at higher growth rates. Furthermore, a fairly narrow set of species dominates the particle composition and growth. For chemical transport modeling and climate assessments, our study may reduce the needed chemical complexity in such models for parametrizing particle growth in the upper troposphere.

Our findings apply to this experiment, with nearly pure isoprene oxidation by OH radicals forming particles. It is important to emphasize that this does not mean that other systems are not driven by reactive uptake of more volatile species. For this case, we conclude that gas-phase production of condensable species governs nearly kinetic particle growth, but other systems may show different behaviors. The dVBS analysis framework provides a method for analyzing growth contributions and particle-phase processing for these systems during steady-state growth.

## Supplementary Material



## Data Availability

Data used in figures in the manuscript will be available online at 10.5281/zenodo.15877275. A Python implementation of basic dVBS analysis and graphing is also available.

## Notes

Explanatory dVBS diagram; Pearson R correlation analysis; volatility parametrization and temperature dependence; dynamic VBS modeling of dimer enhancement effects; assessment of I^–^-CIMS organonitrate sensitivity; discussion of SVOC uptake behavior; and fitting of growth rate from multiphase measurements (PDF)

## ##

Department of Chemistry and Biochemistry, University of North Carolina—Wilmington, Wilmington, North Carolina 28403, United States.
