# Supplementary material for: Isoprene Aerosol Growth in the Upper Troposphere: Application of the Diagonal Volatility Basis Set to CLOUD Chamber Measurements
Source: ACS EST Air. 2025 Sep 15;2(10):2092–104. doi: 10.1021/acsestair.5c00106 (PMC12623003; doi:10.1021/acsestair.5c00106)
Supplement: Supplementary file 1 [file ea5c00106_si_001.pdf]

Supporting Information: Isoprene Aerosol Growth in the Upper Troposphere: Application of the Diagonal Volatility Basis Set to CLOUD Chamber Measurements

Nirvan Bhattacharyya,[*,†,‡] Brandon Lopez,[†,¶] Jenna DeVivo,[†,‡] Douglas M. Russell,[§] Jiali Shen,[‖,⊥] Eva Sommer,[#,@] João Almeida,[#,△] Antonio Amorim,[△] Hannah M. Beckmann,[▽] Mattia Busato,[#] Manjula R. Canagaratna,[††] Lucia Caudillo-Plath,[§] Anouck Chassaing,[‡‡] Theodoros Christoudias,[¶¶] Lubna Dada,[§§] Imad El-Haddad,[§§] Richard C. Flagan,[‖‖] Hartwig Harder,[⊥⊥] Bernhard Judmaier,[##] Milin Kaniyodical Sebastian,[@@] Jasper Kirkby,[#,§] Hannah Klebach,[§] Markku Kulmala,[‖,⊥] Felix Kunkler,[⊥⊥] Katrianne Lehtipalo,[‖,△△] Lu Liu,[§§] Bernhard Mentler,[##] Ottmar Möhler,[@@] Aleksandra Morawiec,[@] Tuukka Petäjä,[‖] Pedro Rato,[#,§] Birte Rörup,[‖] Samuel Ruhl,[⊥⊥] Wiebke Scholz,[##] Mario Simon,[§] António Tóme,[▽▽▽] Yandong Tong,[†††,‡‡‡] Jens Top,[§§] Nsikanabasi Silas Umo,[@@] Rainer Volkamer,[†††,‡‡‡] Jakob Weissbacher,[##] Doug R. Worsnop,[‖,††] Christos Xenofontos,[¶¶] Boxing Yang,[§§] Wenjuan Yu,[‖] Marcel Zauner-Wieczorek,[§] Imad Zgheib,[¶¶¶] Jiangyi Zhang,[‖] Zhensen Zheng,[##,§§§] Xu-Cheng He,[‖,‖‖‖] Dominik Stolzenburg,[⊥⊥⊥] Siegfried Schobesberger,[###] Joachim Curtius,[§] and Neil M. Donahue[*,†,‡,¶,@@@]

†*Center for Atmospheric Particle Studies, Carnegie Mellon University, Pittsburgh, PA 15213, USA*
‡*Department of Chemistry, Carnegie Mellon University, Pittsburgh, PA 15213, USA*
¶*Department of Chemical Engineering, Carnegie Mellon University, Pittsburgh, PA 15213, USA*
§*Institute for Atmospheric and Environmental Sciences, Goethe University Frankfurt, Frankfurt am Main 60438, Germany*
‖*Institute for Atmospheric and Earth System Research/Physics, Faculty of Science, University of Helsinki, Helsinki 00014, Finland*
⊥*Helsinki Institute of Physics, University of Helsinki, Helsinki 00014, Finland*
#*CERN, the European Organization for Nuclear Research, Geneva 1211, Switzerland*
@*Faculty of Physics, University of Vienna, Wien 1010, Austria*
△*CENTRA and Faculdade de Ciências da Universidade de Lisboa, Lisboa 1749-0016, Portugal*
▽*Department of Environmental Physics, University of Tartu, Tartu 50090, Estonia*
††*Aerodyne Research, Inc., Billerica, MA 01821, USA*
‡‡*Department of Environmental Science, Stockholm University, Stockholm 10691, Sweden*
¶¶*Climate and Atmosphere Research Centre, The Cyprus Institute, Nicosia 1645, Cyprus*
§§*PSI Center for Energy and Environmental Sciences, Villigen PSI 5232, Switzerland*
‖‖*Division of Chemistry and Chemical Engineering, California Institute of Technology, Pasadena, CA 91125, USA*
⊥⊥*Max Planck Institute for Chemistry, Mainz 55128, Germany*
##*Institute for Ion Physics and Applied Physics, University of Innsbruck, Innsbruck 6020, Austria*
@@*Institute of Meteorology and Climate Research, Karlsruhe Institute of Technology, Karlsruhe 76131, Germany*
△△*Finnish Meterological Institute, Helsinki 00560, Finland*
▽▽▽*Instituto Dom Luiz, Universidade da Beira Interior, Covilhã 6201, Portugal*
†††*Department of Chemistry, University of Colorado Boulder, Boulder, CO 80309, USA*
‡‡‡*Cooperative Institute for Research in Environmental Sciences, University of Colorado Boulder, Boulder, CO 80309, USA*
¶¶¶*Tofwerk AG, Thun 3645, Switzerland*
§§§*IONICON Analytik GmbH, Innsbruck 6020, Austria*
‖‖‖*Yusuf Hamied Department of Chemistry, University of Cambridge, Cambridge CB21TN, United Kingdom*
⊥⊥⊥*Institute of Materials Chemistry, TU Wien, Vienna 1040, Austria*
###*Department of Technical Physics, University of Eastern Finland, Kuopio 70211, Finland*
@@@*Department of Engineering and Public Policy, Carnegie Mellon University, Pittsburgh, PA 15213, USA*

E-mail: nirvanb@andrew.cmu.edu; nmd@andrew.cmu.edu

S1 Diagonal Volatility Basis Set Diagram

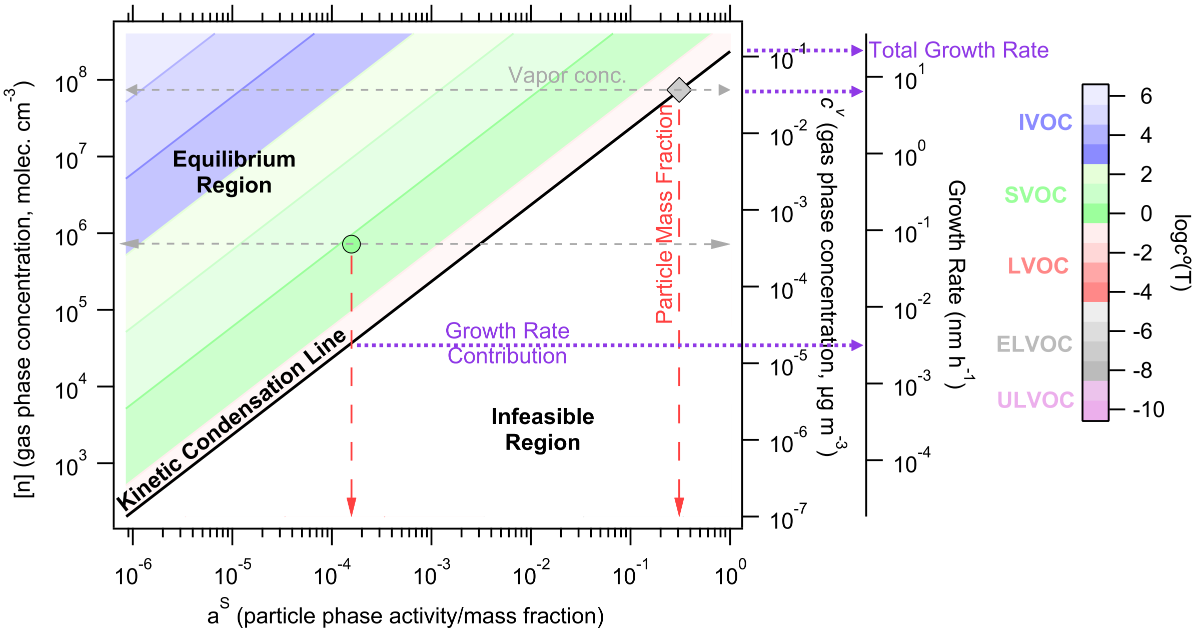

Figure S1: Diagonal volatility basis set figure with a growth rate of 21.4 nm h^{-1} showing an ELVOC species on the kinetic condensation line and an SVOC species in the equilibrium space. Infeasible region is labeled. Points are colored according to their volatility. Diagonal bands of color shown in the equilibrium region of the graph correspond to compound volatility classes. Gray horizontal lines indicate where to read the gas phase concentration of each point on the y-axes, red vertical lines show the particle phase mass fraction on the x-axis, and the purple dashed arrows indicate growth rate contributions. Total growth rate is indicated by reading across to the growth rate axis from the right y-intercept of the kinetic condensation line (when $a^s = 1$). Growth rate contributions for individual compounds are read by reading down to the kinetic condensation line and then across to the growth rate axis. For kinetically condensing species, you simply read directly across.

S2 Correlation Analysis for Low-Concentration Gas Phase Compounds

The nominal limit of detection of the gas phase mass spectrometer measurements is approximated as 10^{-4} μg m^{-3} (3×10^5 cm^{-3}) in this work. While this LOD varies across each instrument and may be compound specific within each instrument, we apply a flat nominal LOD in this analysis. For species with gas phase concentrations in run 2620.14 below the nominal LOD, we perform Pearson correlation coefficient (R) analysis between these species and representative tracers. For compounds with no nitrogen, $C_5H_{12}O_5$ and $C_5H_{12}O_6$ are used as oxidation tracers, while for nitrogen containing compounds, $C_5H_{11}O_4NO_3$ and $C_5H_{11}O_5NO_3$ are used. This correlation analysis compares gas phase data across the whole CLOUD16 isoprene oxidation run and rejects any compounds with R<0.5 for both of its tracer species. Note that the Pearson correlation coefficient only assesses linear correlations and non-linear behaviors are not captured. Furthermore, we only consider a positive correlation test because these species are all later generation oxidation products. Compounds which pass this test are included in the presented data because they respond to the variation in

the chamber even if their nominal concentration during the experiment in question is below the nominal LOD.

S3 Volatility Parameterization and Temperature Dependence

The history of the volatility basis set (VBS), its application, and the improvements made to its relevant volatility parameterizations and temperature dependence has been discussed thoroughly in Stolzenburg et al. (2022).[S1] In this study, the primary method for calculating the appropriate volatility of any given compound is the parameterization for the saturation concentration at 300K (c°[300K]) based on its carbon, oxygen, and nitrogen content. The functional form of this equation is shown in Equation 1, where $n_{C/O/N}$ represents the number of carbons, oxygens, and nitrogens in a given compounds, while n_C^0 and the b_i terms are empirical constants of the parameterization. For this study, the values from Stolzenberg et al.(2022)[S2] are used. So here, n_C^0 is 25, while b_C, b_O, b_{CO}, b_N are 0.475, 1.4, -0.3, and 2.5, respectively.

$$\log_{10} c^0(300K) = \left(n_C^0 - n_C\right) b_C - \left(n_O - 3n_N\right) b_O - 2\frac{n_C\left(n_O - 3n_N\right)}{n_C + n_O - 3n_N} b_{CO} - n_N b_N \tag{1}$$

This elemental approach is motivated by the constraints of typical gas and particle phase mass spectrometry, which can only yield elemental composition information rather than detailed molecular structures. For isoprene oxidation, observational and mechanistic background provides insight into the the molecular formula and functionalization of some isoprene oxidation products.[S3,S4] Where we can assign a reasonable molecular structure, we use the SIMPOL group contribution method to assess the volatility of those compounds.[S5] For example, the compound $C_5H_{12}O_6$ has been observed in isoprene oxidation studies to be a 5 member carbon backbone with 2 hydroperoxy (-OOH) groups and 2 hydroxy (-OH) groups, which can be input into the SIMPOL model to extract the c°[300K].

Both volatility parameterization methods estimate a saturation concentration at 300K, not the frigid temperature used in these experiments. Epstein et al. (2010)[S6] outlined the appropriate adjustment using a Clausius-Clapeyron treatment as shown in Equation 2. This temperature dependence is related to the enthalpy of vaporization (ΔH_{vap}).

$$\log_{10} c^0(T) = \log_{10} c^0(300K) + \frac{1000 \cdot \Delta H_{\text{vap}}}{R \cdot \ln(10)} \left(\frac{1}{300} - \frac{1}{T}\right) \tag{2}$$

The enthalpy of vaporization can be empirically related to saturation vapor concentration by Equation 3, where a and b are empirical fit parameters. In this study, we use the fit parameters from Stolzenberg et al (2018),[S2] where $a = -5.7$ and $b = 129$.

$$\Delta H_{\text{vap}} \left[\frac{\text{kJ}}{\text{mol K}}\right] = a \cdot \log_{10} c^0(300K) + b \tag{3}$$

S4 Dynamic Volatility Basis Set Modeling of Dimer Enhancement

The dimer enhancement observed in the diagonal volatility basis set may be due to high concentrations of dimers in the smallest nucleating clusters and particles. We examine this by modeling the nucleation and growth of a particle population using a dynamic volatility basis set model as

described in Stolzenberg et al. (2022).[S1] We use the example volatility distribution from Lopez et al. (2025)[S7] which spans the full range of saturation concentrations or volatility classes from ULVOC to IVOC categories. The model is initiated with 1 nm particles at a concentration of 1000 cm^{-3} growing at 10 nm h^{-1}. Nucleation occurs at 10 cm^{-3} s^{-1} and the singular nucleating species is the lowest volatility ULVOC class which acts as a dimer proxy. The simulation runs until the mean mass mode of the particle distribution is approximately 20 nm. The simulated diagonal VBS at the end of this growth period is shown in Figure S2. VBS categories appear as expected, with ULVOCs, ELVOCs, and most LVOCs falling on the kinetically limited condensation line, while equilibrium species reside within their volatility color categories.

The lowest volatility ULVOC point does deviate from the kinetic diagonal and makes a break for the forbidden region. This rightward shift is small small, on the order of 10^{-4} and represents the total predicted absolute increase in nucleating dimers. This early dominance has a minimal impact on overall particle mass given that mass goes as the cube of particle diameter. In the real case, such a shift would be spread across a range of dimer/nucleating species and would cause minimal total rightward movement. We conclude that observed dimer enhancement is most likely related to instrumental limitations of our suite of mass spectrometers with high uncertainties in these gas phase concentrations below the nominal LOD and FIGAERO data near its particle phase LOD.

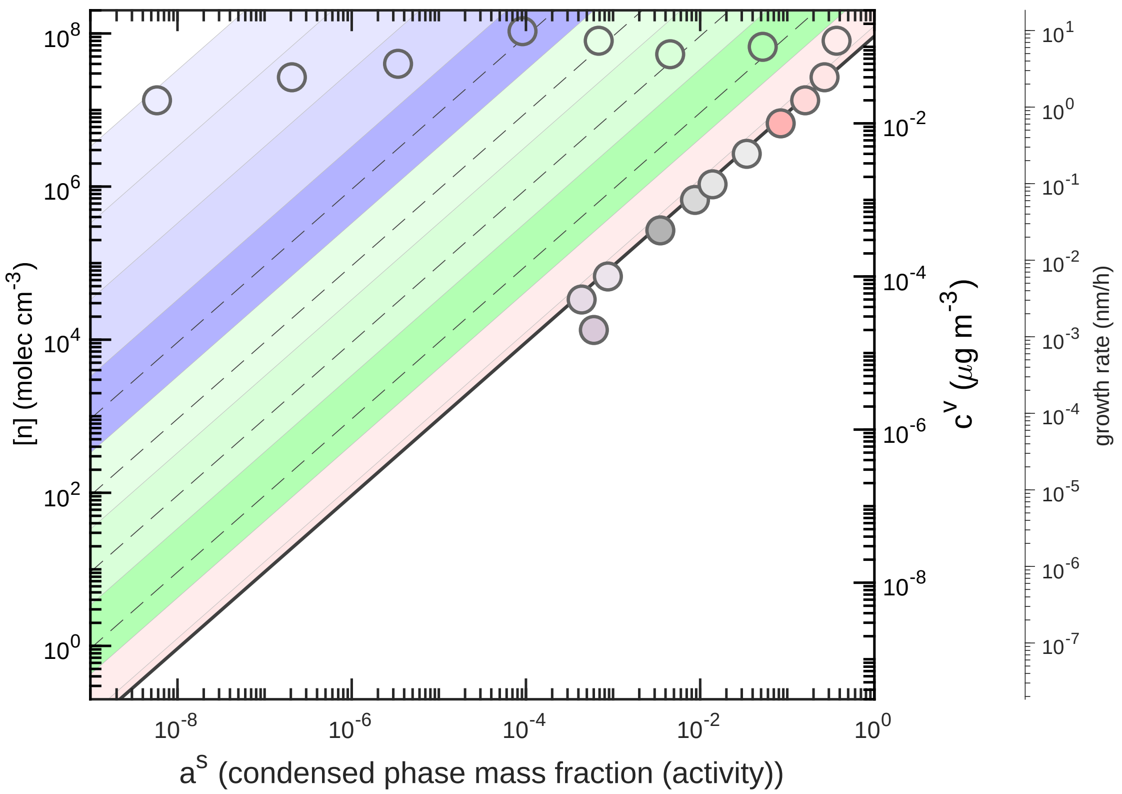

Figure S2: Diagonal volatility basis set simulated from dynamic modeling of aerosol nucleation and growth using the dynamic VBS model described in Stolzenberg et al. (2022).[S1] Slight enhancement of the nucleating ULVOC species is observed.

S5 I^--CIMS Sensitivity to Organonitrate IP-OOMS

Sensitivity of the I^--CIMS varies across orders of magnitude with compound identity.[S8] Direct calibration of the I^--CIMS is difficult due to a lack of available standards for many species. No direct calibration of instrument signal response was performed in this study. However, the instrument was measuring the gas composition of the CLOUD chamber simultaneously with well-calibrated mass spectrometers. These measurement can be used to aid I^--CIMS quantification. NO_3^--CIMS and Br-MION2-CIMS are both quantified using sulfuric acid calibration during the CLOUD campaign which provides a lower bound on all species concentrations with the approximate factor 2 uncertainty in sulfuric acid calibration factor.[S9]

In Figure S3A, the gas-phase signal in the I^--CIMS is compared to quantified gas-phase concentration during the experimental run (2620.14) under study. Even at a similar gas-phase concentrations, several gas-phase species have a lower I^--CIMS signal by several orders of magnitude. In Figure S3B, we use gas-phase measurements from the I^--CIMS over a 2-day experimental period to generate compound specific instrumental sensitivity assuming NO_3^--CIMS and Br-MION2-CIMS measurements are accurate. For species with sufficient gas phase data above the limit of detection across multiple instruments, I^--CIMS gas-phase sensitivity spans over 5 orders of magnitude. The two organonitrate species available in this data set have sensitivities at least 1 order of magnitude lower than the least sensitive non-nitrate IP-OOM. The average sensitivity of monomer non-nitrate IP-OOMS is 1800 cps per μg m^{-3}, while the organonitrates have sensitivities ranging from 8 to 14 cps per μg m^{-3}. Both gas- and particle-phase samples go to the same humidified ion-molecule reactor in the I^--CIMS and so we assume sensitivity differences measured in the gas phase are mirrored in the particle phase. In this study, to avoid excessive autocorrelation between gas and particle phase measurements, we simply enhance FIGAERO-CIMS organonitrate signals in the particle phase by a factor of 30 to account for this relative sensitivity difference. We capture the uncertainty in this relative sensitity by showing error bars extending from a factor 10 to a factor 100 increase in organonitrate signal. Future assessments need more tightly explicit calibration of the FIGAERO-CIMS and additional particle composition measurements.

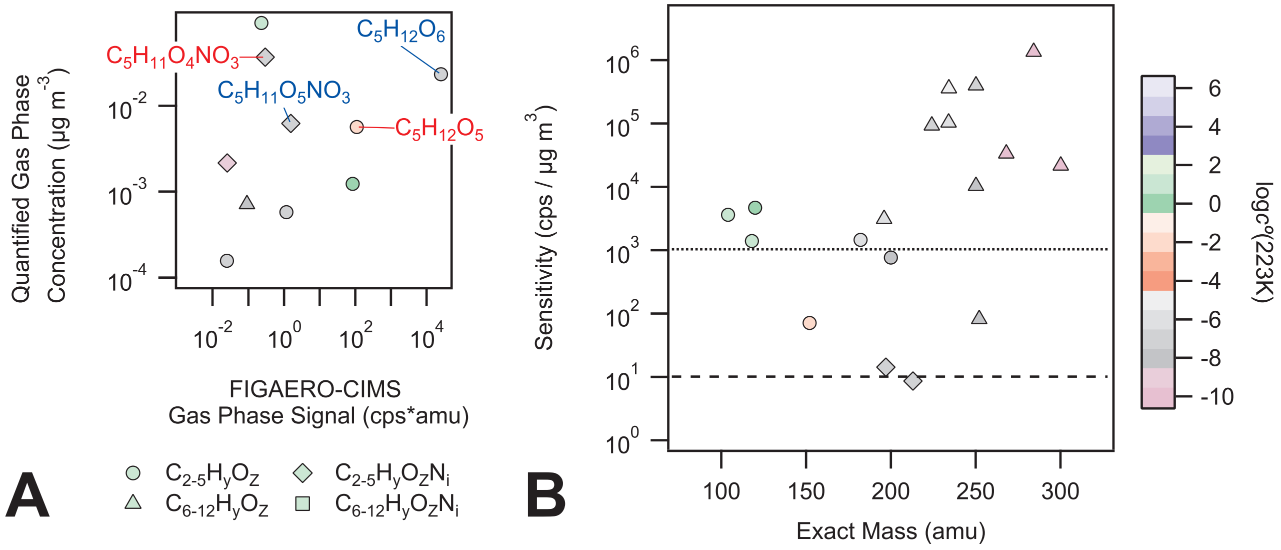

Figure S3: (A) Gas-phase concentrations from other mass spectrometers compared to I^--CIMS arbitrary signal for run 2620.14 experiment. Hydrolysis pairs of interest are labeled. (B) I^--CIMS gas phase sensitivity against compound mass across a 2-day experimental period. Sensitivity derived from comparison to gas-phase mass spectrometer data. Dashed lines indicate approximate sensitivity of organonitrate (large dashes) and non-nitrate (small dashed) monomers, which differ by approximately 2 orders of magnitude.

S6 SVOC uptake inhibition and reactive uptake

As discussed in the main text, the position of the SVOC species $C_5H_{10}O_3$ in the dVBS is shifted left by a factor of 50. This could be explained by diffusion limitations reducing uptake of SVOC species. Uptake to the particle may be inhibited by slow diffusion into the bulk, which generates a surface film with increased activity and subsequently reduces net flux to the particle. In the dVBS, the effect should be minimal for kinetically condensing species but should shift equilibrium species to the left and potentially out of their volatility bin/sub-bin.

In Figure S4, we recreate Figure 3 from the main text with the addition of red bordered star symbols showing the position of the 2 SVOC species which are detected in the gas phase, but undetected in the particle phase. Their position on the y-axis is determined by their measured gas phase concentration, while their x-coordinate is their expected equilibrium particle phase contribution reduced by a factor of 50 to simulate uptake inhibition. These species, which would have fallen near the FIGAERO limit of detection, are well below this limit if condensational uptake was inhibited by diffusion limitations.

The other SVOC species detected in both phases, $C_4H_8O_3$ and $C_4H_8O_4$, are labeled on Figure S4. Both of these species are enhanced rightward and fall directly on the condensation line, indicating that their effective uptake coefficients are near unity. However, both species have peak thermal desorption temperatures (T_{max}) between 50 and 70°C, not consistent with SVOC species. Desorption temperatures in this range are similar to those observed for ELVOC species like $C_5H_{12}O_6$. If their parameterized volatilties were to be taken at face value, both species must undergo reactive uptake and thermal decomposition to explain such unity uptake. If their T_{max} derived volatility is accurate, then both species arrive on the condensation line as expected.

As discussed in the main text, these divergent SVOC uptake processes may be consistent. Diffusion limitations impacting equilibration of SVOC species with the particle bulk do not necessarily

impact reactive processes occurring near the surface of the particle. However, none of the observed processes impact particle growth behavior and in general do not provide strong enough evidence for either uptake inhibition or reactive uptake processes impacting equilibrium products of isoprene oxidation.

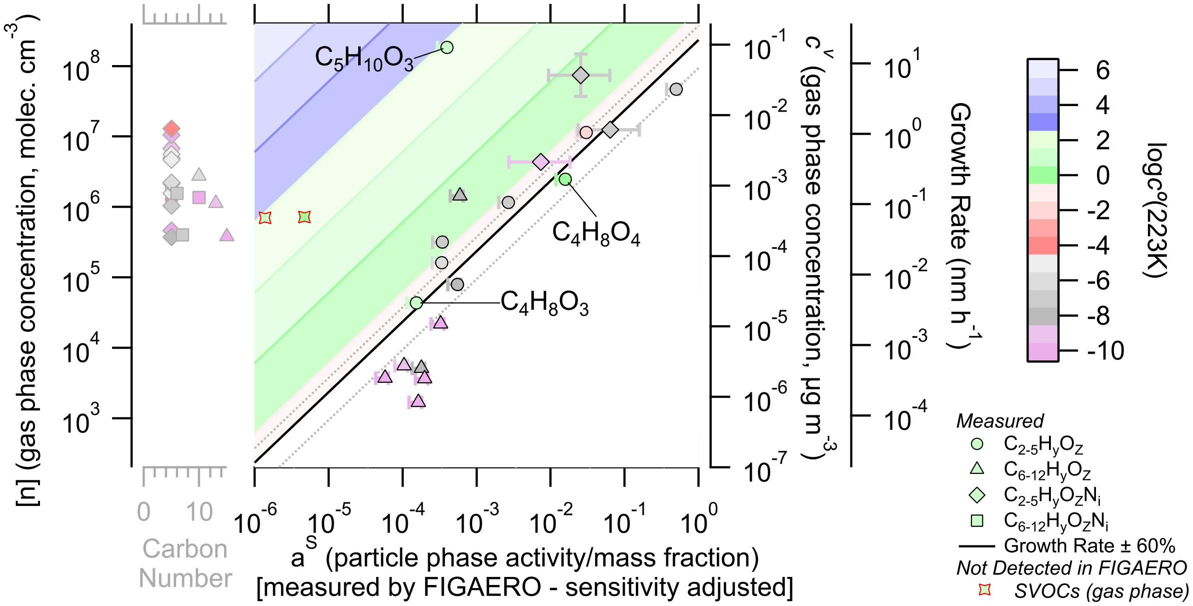

Figure S4: dVBS representation similar to Figure 3 in the main text with the addition of labels for detected SVOCs and red-bordered stars representing SVOC species which were detected in the gas phase, but not in the particle phase. The particle mass fraction of the stars is their equilibrium position reduced by a factor of 50.

S7 Growth rate prediction from multiphase measurements

Growth rates can be determined based on the gas and particle phase measurements by finding the best-fit condensation line in the space. We generate this line by numerically sweeping the condensational line across the space from GR = 0.01 to 100 nm h^{-1} and calculating the sum of the logarithmic orthogonal distance from each point to the line. This sum may be weighted by the volatility using the gamma function described in the main text. Results from this fitting procedure are showing in Figure S5.

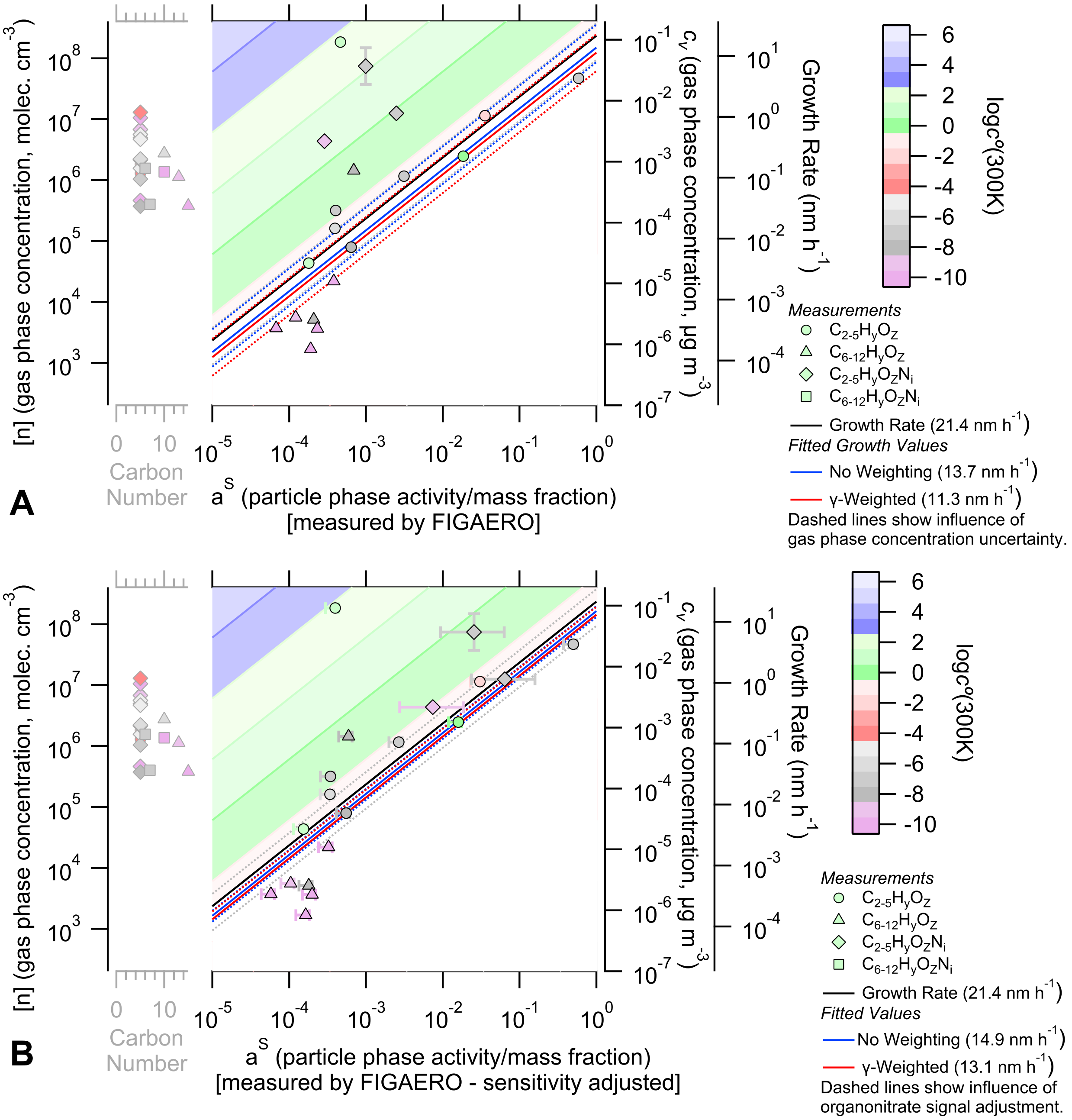

Figure S5: Presentation of growth rate prediction for run 2620.14. (A) dVBS for gas and particle measurements without adjustment for FIGAERO organonitrate sensitivity (as in Figure 2). Growth rate fitted by minimizing total logarithmic orthogonal distance is presented. Distances are unweighted in fits shown in blue and weighted by volatility using the gamma function for fits shown in red. Dashed lines show how fitted growth rate shifts as gas phase measured concentrations vary by a factor of 2. (B) dVBS for gas and particle measurements with a factor 30 adjustment to particle phase organonitrate signal (as in Figure 3). Distances are unweighted in fits shown in blue and weighted by volatility using the gamma function for fits shown in red. Dashed lines show how fitted growth rate shifts as organonitrate signal is adjusted by a factor of 10 or 100.

References

(S1) Stolzenburg, D.; Wang, M.; Schervish, M.; Donahue, N. M. Tutorial: Dynamic organic growth modeling with a volatility basis set. *Journal of Aerosol Science* **2022**, *166*, 106063.

(S2) Stolzenburg, D.; Fischer, L.; Vogel, A. L.; Heinritzi, M.; Schervish, M.; Simon, M.; Wagner, A. C.; Dada, L.; Ahonen, L. R.; Amorim, A.; Baccarini, A.; Bauer, P. S.; Baumgartner, B.; Bergen, A.; Bianchi, F.; Breitenlechner, M.; Brilke, S.; Mazon, S. B.; Chen, D.; Dias, A.; Draper, D. C.; Duplissy, J.; Haddad, I. E.; Finkenzeller, H.; Frege, C.; Fuchs, C.; Garmash, O.; Gordon, H.; He, X.; Helm, J.; Hofbauer, V.; Hoyle, C. R.; Kim, C.; Kirkby, J.; Kontkanen, J.; Kürten, A.; Lampilahti, J.; Lawler, M.; Lehtipalo, K.; Leiminger, M.; Mai, H.; Mathot, S.; Mentler, B.; Molteni, U.; Nie, W.; Nieminen, T.; Nowak, J. B.; Ojdanic, A.; Onnela, A.; Passananti, M.; Petäjä, T.; Quéléver, L. L.; Rissanen, M. P.; Sarnela, N.; Schallhart, S.; Tauber, C.; Tomé, A.; Wagner, R.; Wang, M.; Weitz, L.; Wimmer, D.; Xiao, M.; Yan, C.; Ye, P.; Zha, Q.; Baltensperger, U.; Curtius, J.; Dommen, J.; Flagan, R. C.; Kulmala, M.; Smith, J. N.; Worsnop, D. R.; Hansel, A.; Donahue, N. M.; Winkler, P. M. Rapid growth of organic aerosol nanoparticles over a wide tropospheric temperature range. *Proceedings of the National Academy of Sciences of the United States of America* **2018**, *115*, 9122–9127.

(S3) Wennberg, P. O.; Bates, K. H.; Crounse, J. D.; Dodson, L. G.; McVay, R. C.; Mertens, L. A.; Nguyen, T. B.; Praske, E.; Schwantes, R. H.; Smarte, M. D.; St Clair, J. M.; Teng, A. P.; Zhang, X.; Seinfeld, J. H. Gas-Phase Reactions of Isoprene and Its Major Oxidation Products. *Chemical Reviews* **2018**, *118*, 3337–3390.

(S4) Curtius, J.; Heinritzi, M.; Beck, L. J.; Pöhlker, M. L.; Tripathi, N.; Krumm, B. E.; Holzbeck, P.; Nussbaumer, C. M.; Hernández Pardo, L.; Klimach, T.; Barmpounis, K.; Andersen, S. T.; Bardakov, R.; Bohn, B.; Cecchini, M. A.; Chaboureau, J. P.; Dauhut, T.; Dienhart, D.; Dörich, R.; Edtbauer, A.; Giez, A.; Hartmann, A.; Holanda, B. A.; Joppe, P.; Kaiser, K.; Keber, T.; Klebach, H.; Krüger, O. O.; Kürten, A.; Mallaun, C.; Marno, D.; Martinez, M.; Monteiro, C.; Nelson, C.; Ort, L.; Raj, S. S.; Richter, S.; Ringsdorf, A.; Rocha, F.; Simon, M.; Sreekumar, S.; Tsokankunku, A.; Unfer, G. R.; Valenti, I. D.; Wang, N.; Zahn, A.; Zauner-Wieczorek, M.; Albrecht, R. I.; Andreae, M. O.; Artaxo, P.; Crowley, J. N.; Fischer, H.; Harder, H.; Herdies, D. L.; Machado, L. A.; Pöhlker, C.; Pöschl, U.; Possner, A.; Pozzer, A.; Schneider, J.; Williams, J.; Lelieveld, J. Isoprene nitrates drive new particle formation in Amazon's upper troposphere. *Nature 2024 636:8041* **2024**, *636*, 124–130.

(S5) Pankow, J. F.; Asher, W. E. SIMPOL.1: A simple group contribution method for predicting vapor pressures and enthalpies of vaporization of multifunctional organic compounds. *Atmospheric Chemistry and Physics* **2008**, *8*, 2773–2796.

(S6) Epstein, S. A.; Riipinen, I.; Donahue, N. M. A semiempirical correlation between enthalpy of vaporization and saturation concentration for organic aerosol. *Environmental Science and Technology* **2010**, *44*, 743–748.

(S7) Lopez, B.; Bhattacharyya, N.; DeVivo, J.; Wang, M.; Caudillo-Plath, L.; Surdu, M.; Bianchi, F.; Brasseur, Z.; Buchholz, A.; Chen, D.; Duplissy, J.; He, X.-C.; Hofbauer, V.; Mahfouz, N.; Makhmutov, V.; Marten, R.; Mentler, B.; Philippov, M.; Schervish, M.; Wang, D. S.; Weber, S. K.; Welti, A.; El Haddad, I.; Lehtipalo, K.; Kulmala, M.; Worsnop, D.; Kirkby, J.; Mauldin, R. L.; Stolzenburg, D.; Schobesberger, S.; Flagan, R.; Donahue, N. M. A diagonal

volatility basis set to assess the condensation of organic vapors onto particles. *Environmental Science: Atmospheres* **2025**,

(S8) Iyer, S.; Lopez-Hilfiker, F.; Lee, B. H.; Thornton, J. A.; Kurtén, T. Modeling the Detection of Organic and Inorganic Compounds Using Iodide-Based Chemical Ionization. *Journal of Physical Chemistry A* **2016**, *120*, 576–587.

(S9) Kürten, A.; Rondo, L.; Ehrhart, S.; Curtius, J. Calibration of a chemical ionization mass spectrometer for the measurement of gaseous sulfuric acid. *Journal of Physical Chemistry A* **2012**, *116*, 6375–6386.
